# Diversity of Antimicrobial Resistance and Virulence Determinants in *Pseudomonas aeruginosa* Associated with Fresh Vegetables

**DOI:** 10.1155/2012/426241

**Published:** 2012-11-20

**Authors:** Kashina Allydice-Francis, Paul D. Brown

**Affiliations:** ^1^Department of Microbiology, Bureau of Standards Jamaica, Kingston 10, Jamaica; ^2^Biochemistry Section, Department of Basic Medical Sciences, University of the West Indies, Mona, Kingston 7, Jamaica

## Abstract

With the increased focus on healthy eating and consuming raw vegetables, this study assessed the extent of contamination of fresh vegetables by *Pseudomonas aeruginosa* in Jamaica and examined the antibiotic susceptibility profiles and the presence of various virulence associated determinants of *P. aeruginosa*. Analyses indicated that vegetables from retail markets and supermarkets were widely contaminated by *P. aeruginosa*; produce from markets were more frequently contaminated, but the difference was not significant. Lettuce and carrots were the most frequently contaminated vegetables, while tomatoes were the least. Pigment production (Pyoverdine, pyocyanin, pyomelanin and pyorubin), fluorescein and alginate were common in these isolates. Imipenem, gentamicin and ciprofloxacin were the most inhibitory antimicrobial agents. However, isolates were resistant or showed reduced susceptibility to ampicillin, chloramphenicol, sulphamethoxazole/trimethoprim and aztreonam, and up to 35% of the isolates were resistant to four antimicrobial agents. As many as 30% of the isolates were positive for the *fpv1* gene, and 13% had multiple genes. Sixty-four percent of the isolates harboured an exoenzyme gene (*exoS*, *exoT*, *exoU* or *exoY*), and multiple exo genes were common. We conclude that *P. aeruginosa* is a major contaminant of fresh vegetables, which might be a source of infection for susceptible persons within the community.

## 1. Introduction

*Pseudomonas aeruginosa* is an oxidase-positive, nonfermentative, motile, and gram-negative bacterium that is ubiquitous and very versatile. While *P. aeruginosa* is considered an opportunistic pathogen, several reports indicate that the organism can also cause infections in healthy hosts [1–4]. Further, evidence has suggested that there are no major differences in virulence between clinical and environmental isolates, for example, clone and pilin-type distributions [5], pilin genes [6], flagellin genes [7], genes for multidrug efflux and type III secretion system, the porin gene *oprD* [8], haemolytic and proteolytic activities, and invasion of epithelial cells [9]. Consequently, consuming raw vegetables that have been contaminated by this organism could have serious implication on human health. 

Although many of the organisms associated with food plants are nonpathogenic, many, including vegetables, may be contaminated through insufficiently-treated water and fertilizers or may be compromised by the use of biocides during cultivation. Contamination can occur in the field, during harvest, processing, distribution, and even at use [10, 11].

The intrinsic and acquired resistance of *P. aeruginosa* to many structurally-unrelated antibiotics is due to several adaptations, including active efflux systems, reduced cell wall permeability, plasmid acquisition, expression of various enzymes, or by biofilm formation [12, 13]. Pathogenesis involves production of both extracellular and cell-associated virulence factors [14]. Many virulence factors are expressed through a cell density-dependent mechanism known as quorum sensing [14]. These additional virulence factors include elastase, lipase, protease, and several cytotoxins, encoded by *exo* genes. Elastase and alkaline protease are known to degrade a large variety of tissue components such as proteinaceous elements of connective tissue and cleave the cell surface receptors on neutrophils [15].

In the present study, we examined the prevalence and antibiotic-resistance profiles of the organism and the presence of various virulence factors in *P. aeruginosa *isolated from vegetables obtained from various markets and supermarkets across Jamaica.

## 2. Materials and Methods

### 2.1. Microbial Isolation and Identification

Seventeen individual retail market and supermarket outlets, including two canteens serving lunches, located in several parishes in Jamaica were selected for the study. A total of 95 vegetable samples including lettuce (*n* = 15), white cabbage (*n* = 15), red cabbage (*n* = 3), carrots (*n* = 15), sweet pepper (*n* = 15), cucumber (*n* = 15), tomatoes (*n* = 15), and mixed vegetable salad (*n* = 2) were collected in plastic sample bags and stored 4°C for a maximum of 48 hr. Approximately seven samples were collected from each outlet. Prior to testing, sample bags were wiped with 70% alcohol, and a sterile knife was used to cut samples in sterile trays. Samples were trimmed of any spoiled parts, and their outer leaves removed. Samples were cut into pieces, and 25 g portions placed into sterile 225 mL Tryptic Soy Broth (TSB). Further decimal dilutions were made using physiological saline, and aliquots were plated onto Cetrimide base agar. Plates were incubated at 42°C for 48 hr, and organisms were purified on blood agar and MacConkey agar plates. Further, gram stain, motility, oxidase, and catalase tests were carried out. The criteria for identifying an isolate as *Pseudomonas aeruginosa *were oxidase positivity, catalase positivity, growth at 42°C, and pigment production [16].* P. aeruginosa* PAO1 and PAOR1 (gifts from B. Iglewski) and ATCC 27853 (gift from R. Khan) were used as the reference strains in all tests completed. *P. aeruginosa *isolates were grown on blood agar and MacConkey agar plates to assess purity and on Mueller-Hinton agar plates to assess pigment production. Pyoverdine and pyocyanin production was assessed on *Pseudomonas* B and *Pseudomonas* A media, respectively. *Escherichia coli* isolates were purified on MacConkey agar and then inoculated on Triple iron agar, Urea agar, Simmons citrate agar, and Motility Indole-lysine media. Isolates that showed similar biochemical reactions to the *E. coli* standard strain ATCC25922 were selected for further study. Unless otherwise noted, bacteria were grown in Luria-Bertani medium (Oxoid, Basingstoke, UK, USA) or in Mueller-Hinton broth or agar (Becton Dickinson, Cockeysville, MD) at 37°C for 18 h.

### 2.2. Antimicrobial Susceptibility Testing

Susceptibility testing was performed by the standard CLSI (formerly known as NCCLS) disk diffusion method [17] using common antipseudomonad antibiotics: ampicillin (10 *μ*g), aztreonam (30 *μ*g), ceftazidime (30 *μ*g), chloramphenicol (30 *μ*g), ciprofloxacin (5 *μ*g), gentamicin (10 *μ*g), imipenem (10 *μ*g), sulfamethoxazole-trimethoprim (1.25/23.75 *μ*g), and tetracycline (30 *μ*g) (BD Biosciences, MD, USA). Inocula were prepared by suspending growth from LB broth to a starting concentration of 5 × 10^5^ cfu/mL. Mueller-Hinton plates were incubated at 35°C for 16 to 18 hours after inoculation with organisms and placement of the disks, and zones of inhibition were measured. 

### 2.3. Multiplex PCR for Detection of Virulence Genes

Eight primers were used to assess the distribution of the Type III secretion (TTS) genes, *exoS*, *exoT*, *exoU, *and *exoY* [18]. Bacteria were grown overnight at 37°C in lauryl tryptose broth, and DNA extracted using the Wizard genomic DNA purification kit (Promega Corp.) according to the manufacturer's instructions. The PCR was set up as follows: 1 *μ*L of DNA template (100 to 200 ng), 1 *μ*L of each PCR primer (IDT, USA), (a final 200 mM concentration of each primer), 10 *μ*L of GoTaq MasterMix (Promega), and 1 *μ*L of sterile water. The negative control contained 1 *μ*L sterile water instead of DNA. The PCR was carried out as follows: initial denaturation at 94°C for 2 min; 36 cycles of 94°C for 30 s, 58°C for 30 s, and 68°C for 1 min; a final extension step at 68°C for 7 min. Reaction products were separated in a 2% agarose gel and stained with 0.5 mg/mL of ethidium bromide.

### 2.4. Multiplex PCR for the Identification of fpvAI, fpvAII, and fpvAIII

Six primers were used for the simultaneous amplification of the different *fpvA* genes, which code for the three types of pyoverdine receptors produced by *P. aeruginosa* [19]. The PCR was set up as follows: 1 *μ*L of DNA template (100 to 200 ng), 1 *μ*L of each PCR primer (IDT), (a final 200 mM concentration of each primer), 10 *μ*L of GoTaq MasterMix (Promega), and 3 *μ*L of sterile water. The negative control contained 1 *μ*L of sterile water instead of DNA. The following conditions were used: initial denaturation at 94°C for 3 min, followed by 30 cycles with denaturation at 94°C for 30 s, annealing at 55°C for 30 s and elongation at 72°C for 30 s, and terminating with a last cycle at 72°C for 10 min. 

### 2.5. Statistical Analysis

Statistical analysis was performed using the *χ* ^2^ test. Differences were considered significant at *P* < 0.05.

## 3. Results

Samples of vegetables from markets (72.3%; range 50–100%) were more frequently contaminated than those from supermarkets (55.6%; range 14–86%). This difference was, however, not significant. Lettuce (89% in supermarkets; 100% in markets; *P* > 0.05) and carrots (67% in supermarkets; 100% in markets; *P* < 0.05) were the most frequently contaminated vegetable (Table 1), while tomatoes had the lowest level of contamination (22% in supermarkets; 50% in markets; *P* < 0.05) with counts of nil to 1.5 × 10^3^ cfu/g. One supermarket in the eastern region had lettuce as the only sample being contaminated by *P. aeruginosa* with a count of >3.0 × 10^2^ cfu/g. With the exception of tomatoes and lettuce, all other samples were contaminated with high levels of *P. aeruginosa*. Both samples of mixed vegetable salad were contaminated, with counts of 2.6 × 10^2^ cfu/g and >6.0 × 10^3^ cfu/g.

 A total of 88 isolates of *P. aeruginosa* and three isolates of *E. coli* were recovered from 95 vegetable samples, and two vegetable salads included in this study (Table 2). Isolates were recovered from all vegetable samples, and several samples showed high viable count of bacteria (2.6 × 10^2^ up to >1.2 × 10^6^ cfu/g). 

**Figure 1:**
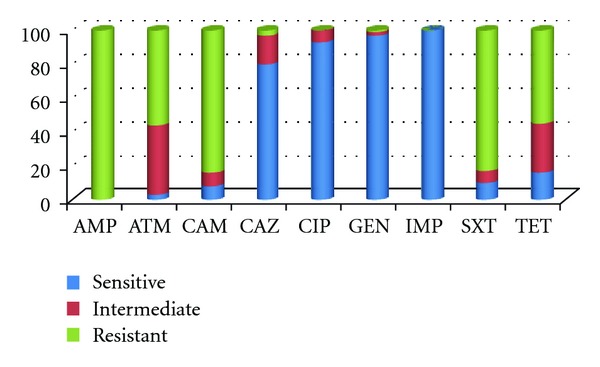
Frequency of resistance among *P. aeruginosa *isolates to common several antimicrobial agents. AMP: ampicillin; ATM: aztreonam; CAM: chloramphenicol; CAZ: ceftazidime; CIP: ciprofloxacin; GEN: gentamicin; IMP: imipenem; SXT: trimethoprim/sulfamethoxazole; TET: tetracycline.

 Imipenem (100%), gentamicin (97%), ciprofloxacin (93%), and ceftazidime (79%) were the most inhibitory antibiotics found in this study based on susceptibility results (Figure 1). However, all isolates were resistant to ampicillin, and 84% and 83% were resistant to chloramphenicol and sulfamethoxazole/trimethoprim, respectively. However, it was of concern that reduced susceptibilities were observed to aztreonam (41%) and tetracycline (29%), given that these are environmental isolates. In the case of these latter antimicrobial agents, these values were in addition to resistance frequencies of 55% observed. Multidrug resistance (defined as resistance to three or more antimicrobial agents) was observed in isolates from all regions: 23%, 35%, and 20% of isolates were resistant to three, four, and five antimicrobial agents, respectively. Two isolates were resistant to six antimicrobials. Both of these isolates were from lettuce samples in the eastern region. Carrots, cabbage, cucumbers, and lettuce were the vegetables frequently associated with multidrug resistant isolates. Resistance to chloramphenicol, ampicillin, and trimethoprim/sulfamethoxazole was most frequently observed.

 Isolates produced pyoverdine, pyorubin, pyocyanin, pyomelanin, and fluorescein pigments. However, it was apparent that more pigmented isolates were obtained from sources in western Jamaica. While some pigments occurred in isolates from all regions (pyoverdine, pyomelanin, and fluorescein), others only occurred in isolates from the central and western (pyorubin) or western and eastern (pyocyanin) regions. In all, 42% isolates produced fluorescein while 68% isolates produced pyoverdine. Several isolates (*n* = 24) were nonpigmented and mucoid due to the overproduction of alginate, and were observed in the all regions, and associated with all vegetable samples. One of the isolates from the vegetable salad was non-pigmented, while the other produced pyoverdine and fluorescein. 

**Figure 2:**
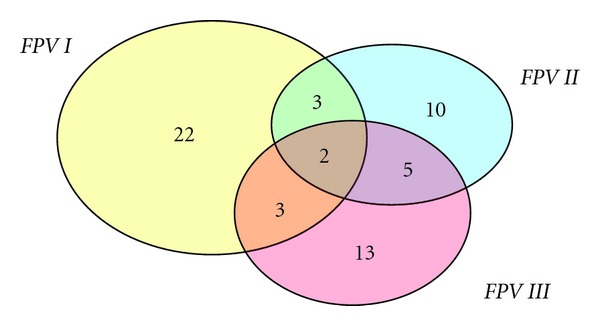
Distribution of pyoverdine receptor genes detected by PCR in *P. aeruginosa* isolates in this study.

 The distributions of the pyoverdine receptor genes are illustrated in Figure 2. While a large proportion of isolates had the *fpvI* gene only (22%), a significant proportion (13%) had multiple genes. The total frequencies of isolates harbouring *fpv1*, *fpv2,* and *fpv3* genes were 30%, 20%, and 23%, respectively. This correlated well with the observation that 68% of isolates produced pyoverdine pigment. Pyoverdine receptor genes were identified in all regions, and multiple genes were associated with cabbage and carrot samples. The two isolates that had all three *FPV* receptors were from cabbage samples: one was from a white cabbage sample from the central region and the other was a red cabbage from the eastern region. 

**Figure 3:**
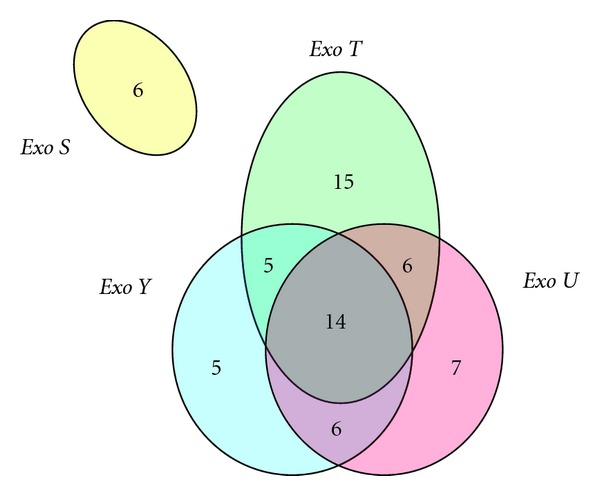
Distribution of exoenzyme genes detected by PCR in *P. aeruginosa* isolates in this study.

 Seventy-three (64%) isolates were PCR-positive for exoenzyme genes: 6% was positive for *exoS* (Figure 3); *exoT*, *exoU,* and *exoY* were identified in 40%, 33%, and 30% of isolates, respectively. While no isolate expressed *exoS* in conjunction with any other exoenzyme gene, 14% of isolates were positive for *exoT*, *exoU,* and *exoY*. These *exo* genes occurred in isolates from all regions, and were more frequently associated with carrots, lettuce, and cabbage samples.

 We identified 19 isolates with multidrug resistance, multiple exoenzyme genes, and at least one *FPV* receptor gene. There was no apparent association noted between pigment production and multiple drug resistance. However, non-pigment producers (alginate producers) were less likely to have genes for exoenzyme or FPV receptor genes, with the exception of two isolates. One was from a red cabbage in the eastern region, which was resistant to five antimicrobial agents, and had *exoT* and *U* and *fpv1*, *fpv2,* and *fpv3*. The other was from sweet pepper in the western region and was resistant to four antimicrobials, had *exoU*, *exoY, fpv2,* and *fpv3*. One isolate from a vegetable salad sample was resistant to four antimicrobials, was pigmented and had *exoT*, *exoU,* and *exoY* in addition to the* fpv3* gene.

## 4. Discussion

Observations showed considerable bacteriological load carried by vegetable samples with total viable counts ranging from 10^2^ to more than 10^6^ cfu/g. The presence of high numbers of viable bacteria, an indicator of the expected shelf life of the vegetable, increases the likelihood of spoilage, as well as the possibility of produce-associated outbreaks [20]. In recent decades, public health promotion of healthier lifestyles has led to increased demand for fresh produce in many countries, including developing countries like Jamaica. Due to the fact that these products are often consumed raw or with very little cooking, consumers face an increased risk of infection from contaminating microorganisms. Despite the risk management strategies instituted in many countries, the number of reported illnesses linked to contaminated produce have increased in the USA [21] and in Europe [22]. While most reports have identified *Salmonella and E. coli* as the main produce-associated pathogens, we have to remain vigilant about the potential risks from others such as *Pseudomonas aeruginosa*. 

 In this study, we sought to identify the prevalence of *P. aeruginosa *contaminating fresh vegetables in markets and supermarkets in Jamaica. We noted that vegetables from both markets and supermarkets were contaminated, with lettuce and carrots being the most frequently contaminated. Numerous studies have investigated the potential sources of contamination in the supply chain at the pre- and postharvest stages. For example, during the preharvest phase, pathogen populations can establish themselves on growing crops, and the risk can be amplified after harvest either by further direct contamination or by proliferation of existing pathogen populations during processing and postharvest handling procedures. Water is likely to be an important source of contamination in the field, including runoff from nearby animal pastures and irrigation from contaminated sources [23, 24]. Further, use of water in postharvest processing has also played a role, for example, in trying to prevent importation of fruit flies [25]. It is well established that pathogens may be disseminated in the environment via the use of inadequately composted or raw animal manures or sewage [26, 27], via the faeces of wild animals [28], or via flies [29, 30]. Post-harvest processes, ranging from storage and rinsing to cutting, are also possible sources of contamination [31], and the use of inadequately decontaminated water in hydrocoolers, which are used to store and process large quantities of fresh produce, can lead to contamination of an entire lot [32]. Given that there were few isolates of *E. coli* relative to *P. aeruginosa*, we concluded that contamination was more likely from nonmanure sources, particularly soil, flies, cockroaches, or rodents. In other words, the organism's presence in the environment and increased temperature and humidity, such as those normally experienced in tropical countries, may be predisposing factors for growth or colonization of vegetables. In fact, frank unsanitary conditions were not observed in the markets included in the study, so it is likely that the growth medium used is selected for *P. aeruginosa* rather than *E. coli*. Further, Shigeharu and coworkers [33] noted that contamination was not markedly decreased after disinfection with sodium hypochlorite, a common agent used to provide protection against pathogenic organisms. Historically, Correa et al. [34] reported that 19% of the fresh vegetable samples fed to oncology patients were found to be contaminated by *P. aeruginosa *even though 1% hypochlorite was used as a disinfectant. Similar to this study, those authors reported that lettuce was among the vegetables that yielded the highest frequency of isolation. Further, the authors reported that some of the pyocin typing and serotyping of vegetable isolates were found to be identical to those recovered from clinical sources.

 Lettuce and carrots were the two vegetables that were mainly associated with *P. aeruginosa* contamination in all regions analyzed in this study. An increased growth of this organism on lettuce may be as a result of its wide surface area. A combination of factors may have contributed to the high contamination yield obtained from all supermarkets and markets across the island as suggested previously. There were no major differences with isolation of *P. aeruginosa *from the various regions assessed as all seem to have been equally implicated by this organism. There was no major difference in counts between vegetables obtained. 

 The inner tissues of vegetables are supposed to be free of microorganisms [35]. For this study, even though the outer leaves were removed from cabbage and lettuce, the recovery of *P. aeruginosa* remained considerably high. The market samples as expected were comparatively more contaminated by *P. aeruginosa* than those of the supermarkets. Considering that poorer persons buy produce in the markets, it is likely that these are at increased risk with the often additional burden of no or inadequate health coverage. In addition to supermarkets and markets, it was considered that consumers may conduct additional washing before use. Through washing, more nutrients become available, and potential pathogenic organisms can spread from contaminated parts to uncontaminated areas [36]. Further, ready-to-eat mixed vegetable salads are frequently contaminated and may be directly related to the quality of the inputs from market or supermarket sources.

 Although healthy individuals require >10^5^ cfu/mL *P. aeruginosa*, the ingestion of <10^3^ cfu/g may colonize the intestine of susceptible individuals and may lead to a gastrointestinal infection, bacteraemia, and haematogenous spread [37]. *Pseudomonas *septicaemia in infants manifested as necrotizing bowel lesions with a history of diarrhoea is also possible [38]. While bacteria in the gastrointestinal tract have been regarded as important in immunocompromised patients, such as those undergoing anticancer chemotherapy, it is of direct concern that the pathogen can also infect persons from the community and might cause fatal bacteraemia [2] and community-acquired pneumonia [1]. Other reports showed incidences of community-acquired *Pseudomonas *infection of the gastrointestinal tract causing diarrhoea in infants [39].

 Further, we noted that several of these isolates had multiple pyoverdine receptors genes. Pyoverdine acts as a siderophore and enables *P. aeruginosa *to acquire more iron from the host cells to carry out its metabolic functions and would therefore increase the virulence of this organism. Pyoverdine also has cytotoxic effect because of its ability to stimulate the production of reactive oxygen and has been determined to be an essential component in biofilm production [14]. Pyocyanin also contributes to the virulence of this organism. Like pyoverdine, it is toxic to both bacterial and eukaryotic cells, this is due to the reactive oxygen that intermediates the organism generates [40]. In a previous study [41], we noted that isolates that produced either pyoverdine or pyocyanin were more likely to produce additional virulence factors. It was therefore not surprising to observe the extent of multiple *exo* genes and/or FPV receptor genes in these organisms. Expression of these genes is governed by quorum sensing [42] and is known to be involved in the pathogenesis of *P. aeruginosa* [43].

 Alginate production by* P. aeruginosa *is an important virulence factor as its expression allows the bacteria to resist phagocytosis. This expression contributes to the development and persistence of the bacteria in patients with chronic pulmonary infections, including cystic fibrosis patients [44–46]. Some of these alginate producing strains had multiple *exo* and *fpv* genes. 

 Many of the isolates were found to have several genes that are expressed and secreted by type 3 secretion systems (TTSS). In this study, we noted that the *exoS* gene did not occur with any other exoenzyme gene and suggest that *exoS* might be mutually exclusive to other *exo* genes. Exoenzyme S inhibits RHO GTPase family signalling, paralyzing macrophages, and inhibiting phagocytosis [47, 48]. *ExoS* and *ExoT* are highly related and have dual functions. *ExoT* contributes to actin cytoskeleton disruption and inhibition of internalization of the bacteria [49, 50]. *ExoU* has the ability to lyse host cell membrane and has cytotoxic effects [51], while *ExoY* acts as adenylate cyclase that increases the levels of intracellular cAMP [52].

*P. aeruginosa *is one of the leading causes of hospital- and community-acquired infections due to its ability to cause a variety of diseases and its high-level resistance to several antibiotics. It was originally believed that this ability was due to the low outer membrane (OM) permeability. However, it is now known to be as a result of the combined action of multidrug resistance pumps in association with OM permeability [53]. However, crop irrigation and application of pesticides with contaminated water can be a primary source of resistant bacteria in a field, and antimicrobial resistance could also originate from bacterial adaptation to heavy metals and plant metabolites, resulting in the formation of multidrug efflux systems [54]. It is also likely that these adaptations could result in increased expression of existing efflux systems. From the isolates studied, many showed multiple resistance to the antimicrobials tested. While most of the isolates were susceptible to ceftazidime, ciprofloxacin, gentamicin, and imipenem, it is of concern that these environmental isolates exhibited significant frequencies of resistance or reduced susceptibilities to so many antimicrobial agents including ampicillin, chloramphenicol, sulfamethoxazole/trimethoprim, tetracycline, and aztreonam. Of note was that many of these isolates expressed pyoverdine pigment and fluorescein and were also alginate-producing strains. While antimicrobial resistance does not appear to be associated with the production of pigment [41], more work might be warranted in environmental isolates, particularly those associated with produce. As has been expressed elsewhere, contaminated vegetables may be an important reservoir or source of community- and hospital-acquired strains of *P. aeruginosa*.

## 5. Conclusions

*P. aeruginosa* contamination of raw vegetables was found to be prevalent in Jamaica as contamination was observed in samples collected from markets and supermarkets in all regions (eastern, western, and central locations). Many of these isolates were resistant or had reduced susceptibilities to multiple antimicrobial agents, produced pigments or alginate, and had multiple exoenzyme or pyoverdine receptor genes. Because of the increased focus on healthy eating and consumption of vegetables, this is a major health concern as the possibility of innocuous spread of pathogenic organisms can be facilitated among unsuspecting and vulnerable individuals. Consequently, the application of proper hygiene practices along the food production/supply chain is essential.

## Figures and Tables

**Table 1:** Level of contamination by *Pseudomonas aeruginosa* of vegetable samples from nine supermarkets and six markets investigated in this study.

Vegetable sample	Supermarket (%)	Markets (%)
Cabbage, white	6/9 (67%)	3/6 (50%)
Cabbage, red	2/3 (67%)	0
Carrots	6/9 (67%)	6/6 (100%)
Cucumbers	5/9 (56%)	4/6 (67%)
Lettuce	8/9 (89%)	6/6 (100%)
Sweet potatoes	5/9 (56%)	3/6 (50%)
Tomatoes	2/9 (22%)	3/6 (50%)

Total	34/57 (60%)	26/36 (72%)

**Table 2:** Frequency of contamination by *Pseudomonas aeruginosa* of vegetable samples from supermarkets, markets, and canteens in the three regions investigated in this study.

Region	Supermarkets	Markets	Canteens
Eastern	11	21	2
Central	14	12	0
Western	16	12	0

Total	41	45	2
